# Supplementary material for: Causal interplay between lactose intolerance and gut microbiota: a combined bidirectional Mendelian randomization and in vivo validation study
Source: Front Nutr. 2026 Jun 1;13:1803337. doi: 10.3389/fnut.2026.1803337 (PMC13265576; doi:10.3389/fnut.2026.1803337)
Supplement: Supplementary file 1 [file Data_Sheet_1.zip › supplementary materials/Forward/forest plot/ebi-a-GCST90027776.finngen_R12_E4_LACTONAS.pdf]

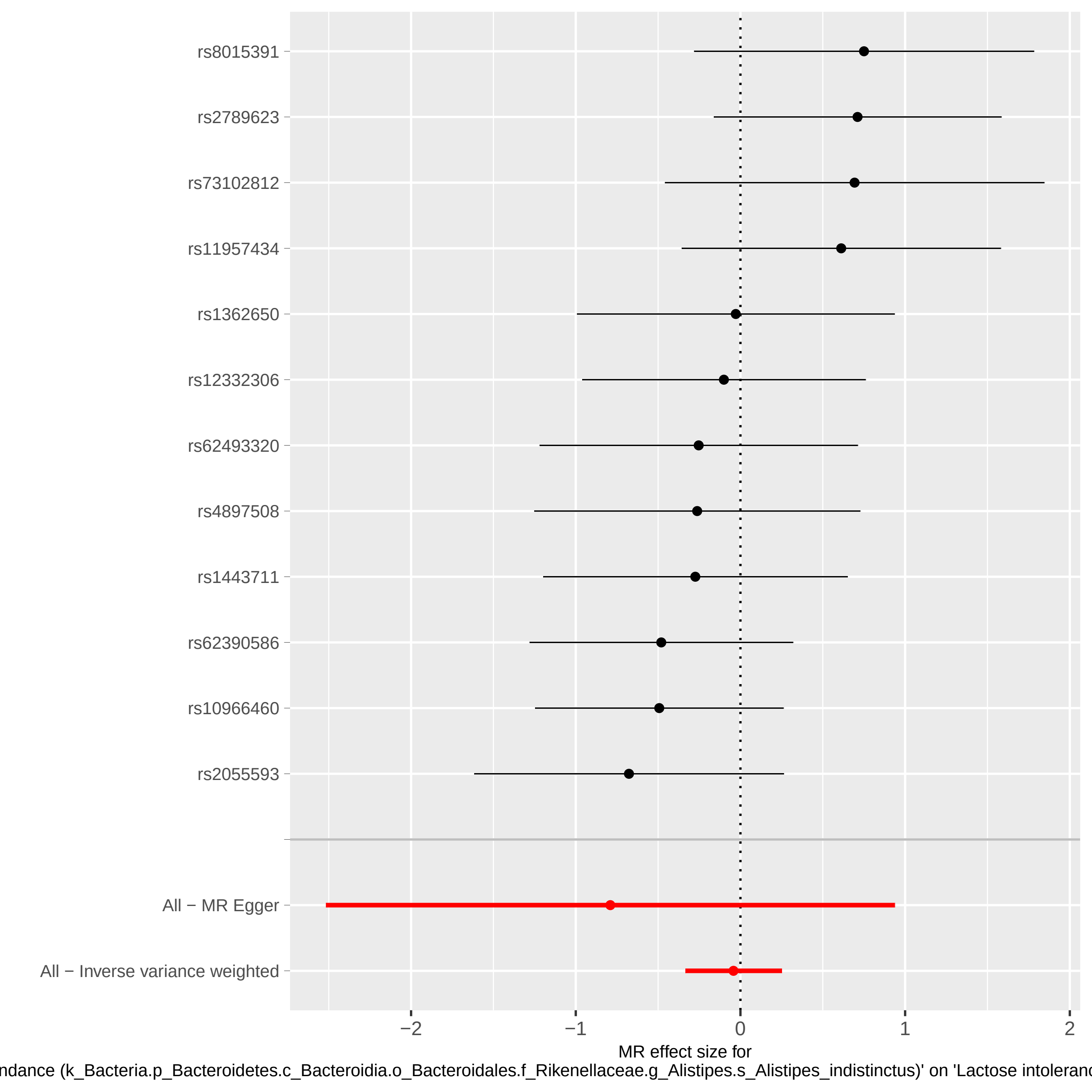
rs8015391
rs2789623
rs73102812
rs11957434
rs1362650
rs12332306
rs62493320
rs4897508
rs1443711
rs62390586
rs10966460
rs2055593
All − MR Egger
All − Inverse variance weighted
−2
−1
0
1
2
MR effect size for
ndance (k_Bacteria.p_Bacteroidetes.c_Bacteroidia.o_Bacteroidales.f_Rikenellaceae.g_Alistipes.s_Alistipes_indistinctus)' on 'Lactose intoleranc
